# Forward and Reverse Signaling Mediated by Transmembrane Tumor Necrosis Factor-Alpha and TNF Receptor 2: Potential Roles in an Immunosuppressive Tumor Microenvironment

**DOI:** 10.3389/fimmu.2017.01675

**Published:** 2017-11-28

**Authors:** Yang Qu, Gang Zhao, Hui Li

**Affiliations:** ^1^Department of Gastrointestinal Cancer Biology, Tianjin Medical University Cancer Institute and Hospital, Tianjin, China; ^2^Key Laboratory of Cancer Immunology and Biotherapy, Tianjin, China; ^3^National Clinical Research Center for Cancer, Tianjin, China

**Keywords:** transmembrane tumor necrosis factor-alpha, TNF receptor 2, tumor microenvironment, forward signaling, reverse signaling

## Abstract

Tumor necrosis factor-alpha (TNF-α) is a pleiotropic inflammatory cytokine produced mainly by activated macrophages, lymphocytes and other cell types. Two distinct forms of TNF-α have been identified: soluble TNF-α (sTNF-α) and transmembrane TNF-α (mTNF-α). mTNF-α, which is the precursor of sTNF-α, can be cleaved by the TNF-α converting enzyme (TACE) and is released as sTNF-α. sTNF-α binds primarily to TNF receptor 1 (TNFR1) and plays an important role in the inflammatory immune response, whereas mTNF-α interacts primarily with TNF receptor 2 (TNFR2) and mediates the promotion of cellular proliferation and survival and other biological effects. It has been reported that the interaction between mTNF-α and TNFR2 induces bi-directional (forward and reverse) signaling in both mTNF-α- and TNFR2-expressing cells. Increasing evidence shows that the forward and reverse signaling mediated by mTNF-α and TNFR2 might play a significant role in the tumor microenvironment. In this review, the role of the crosstalk between mTNF-α and TNFR2 in the tumor microenvironment will be discussed.

Tumor necrosis factor-alpha is also known as cachexin or cachectin and is a potent inflammatory cytokine produced mainly by activated macrophages, lymphocytes, and other cell types (1, 2). It was first demonstrated that serum from bacillus Calmette–Guérin (BCG)-infected mice treated with lipopolysaccharide (LPS) could cause hemorrhagic necrosis in tumors in animals; this effect was mediated by a “tumor-necrotizing factor” (3). In the following years, more details about the signaling pathways triggered by TNF-α and the functions of TNF-α were revealed.

Although TNF-α was first described as a soluble molecule that induces hemorrhagic necrosis in tumor tissues in experimental animals, the following studies reported that TNF-α exerts anti-tumor effects and pro-tumor effects in different circumstances. It has been demonstrated that there are two different forms of this type of cytokine, soluble TNF-α (sTNF-α) and transmembrane TNF-α (mTNF-α) (4, 5). mTNF-α, the precursor of soluble TNF-α, can be cleaved by TACE and released as sTNF-α. Both forms of TNF-α can bind to TNFR1 or TNFR2 and exert pleiotropic effects on various cell types (6). TNFR1 is expressed on most cell surfaces and mediates cytotoxic effects and pro-inflammatory and pro-apoptotic effects, whereas TNFR2 is expressed primarily on lymphocytes and is involved in the activation and proliferation of lymphocytes (7–9). It has been reported that sTNF-α binds primarily to TNFR1 and plays an important role in inflammatory immune responses (10). mTNF-α, however, interacts primarily with TNFR2 (both soluble and transmembrane forms) and mediates effects that are overlapping and contrary to those of sTNF-α (7, 11). Notably, the crosstalk between mTNF-α and TNFR2 triggers bi-directional signaling in target cells and mTNF-α-expressing cells (12–14). Increasing evidence has shown that in the tumor microenvironment, the interaction between mTNF-α and TNFR2 plays a significant role in tumor progression (15). The expression of mTNF-α, its signaling pathway and the biological effects triggered by the interaction between mTNF-α and TNFR2 will be discussed in this review.

## mTNF-α Expressed on Distinct Cell Types Exerts Different Effects

In the 1980s, the gene coding for TNF-α was cloned and expressed by different teams who used different methods; their results marked profound progress in TNF-α research (16–19). In 1988, Kriegler et al. (20) announced that they had identified and characterized a novel, rapidly inducible cell surface cytotoxic integral transmembrane form of TNF-α that could explain the complex physiology of the molecule. mTNF-α is a stable homotrimer and is the precursor form of sTNF-α. mTNF-α can be cleaved by TNF-α converting enzyme (TACE) and then released as sTNF-α into the circulation to exert its biological function *via* type 1 and 2 TNF-α receptors (6). The two forms of TNF-α functions in two fundamentally different ways. mTNF-α is mainly expressed on monocytes/macrophages, lymphocytes, and some other cell types. In addition, mTNF-α acts as a bipolar molecule that transmits signals both as a ligand and as a receptor in a cell-to-cell contact-mediated manner, which means that mTNF-α not only mediates the forward signals to the target cells through cell-to-cell contact but also transmits the reverse (outside-to-inside) signals back into the mTNF-α-bearing cells (5, 21).

As a ligand, mTNF-α expressed on monocytes/macrophages and lymphocytes exhibits stronger cytotoxic activity than sTNF-α, because it is cytotoxic not only to sTNF-α-sensitive target cells but also to sTNF-α-resistant target cells (22, 23). mTNF-α expressed on T cells mediates the host defense against intracellular pathogens and the activation of endothelial cells and B cells and contributes to monocyte cytokine production (5). mTNF-α on dendritic cells can enhance the proliferation and cytotoxic activity of NK cells (24, 25). Activated CD8^+^ T cells express mTNF-α and expand Vβ5^+^ regulatory T cell populations through the transduction of signaling *via* TNFR2 on the Tregs (26). Moreover, some mTNF-α-bearing tumor cells can recruit immunosuppressive cells to the tumor microenvironment *via* the interaction between mTNF-α and TNFR2 to facilitate the evasion of tumor cells (27). On the other hand, as a receptor, mTNF-α mediates reverse signals back into the mTNF-α-bearing cells, such as T cells, monocytes/macrophages, and NK cells, to regulate the immune responses of these different cell types (5). mTNF-α-bearing tumor cells are stimulated by TNFR2 to induce reverse signaling to activate the NF-κB pathway, which can promote tumor cell survival and apoptosis resistance (21, 28). In summary, the current studies indicate that mTNF-α is expressed on monocytes/macrophages, lymphocytes, and even tumor cells and exerts different effects through its interaction with TNFR2.

## The Crosstalk Between mTNF-α-Bearing Cells and TNFR2-Expressing Cells Exerts Distinct Effects on the Tumor Microenvironment

Increasing evidence shows that in the tumor microenvironment, the interaction between mTNF-α and TNFR2 plays a significant role in tumor progression. However, the effects and mechanisms of mTNF-α/TNFR2 interaction in the tumor microenvironment are not identical. On the one hand, it has been reported that the mTNF-α/TNFR2 interaction could promote the progression of cancer by recruiting immunosuppressive cells to the tumor microenvironment or by enhancing survival, metastasis, and apoptosis resistance of tumor cells (15, 21, 27, 28). On the other hand, as mentioned above, the interaction between mTNF-α and TNFR2 causes cytotoxicity toward not only sTNF-α-sensitive target cells but also sTNF-resistant tumor cells (28).

### mTNF-α/TNFR2 Interaction Promotes Immunosuppressive Cell Accumulation in Tumor Microenvironments

Myeloid-derived suppressor cells (MDSCs) are important immunoregulatory cells in the cancer microenvironment. MDSCs are a heterogeneous group of immune cells from the myeloid lineage; they include immature precursors of macrophages, granulocytes, and dendritic cells. MDSCs are characterized by Gr1 and CD11b expression on the cell surface in mice, while in humans, they are identified as HLA^−^DR^−^ CD11b^+^CD33^+^ cells (27, 29). MDSCs possess strong immune suppressive activity, which defines their functions in modulating the immune response and immune tolerance. The expansion and suppressive functions of MDSCs are relevant to chronic inflammatory conditions, especially in neoplastic disorders. The spectrum of action of MDSC activity encompasses that of T cells, NK cells, dendritic cells, and macrophages, which explains the ability of MDSCs to facilitate tumor evasion (8).

Tumor necrosis factor-alpha/TNF receptor 2 signaling is involved in the regulation of recruitment, differentiation, and suppressive activities of this cell population (29). Previous studies have shown that in tumor-bearing mice, multiple inflammatory mediators, including interleukin-1β (IL-1β), IL-6, and prostaglandin E2 (PEG2), produced by tumor cells induce the accumulation of MDSCs in the tumor microenvironment of bone marrow (9, 30, 31). It has been identified that TNFR2^+^ MDSCs are recruited to tumor sites, and in addition to inflammatory factors, mTNF-α expressed by tumor cells can also promote MDSC accumulation *via* TNFR2 expressed by MDSCs (15, 27, 32). Further, in a mouse model implanted with breast cancer 4T1 cells expressing an uncleavable mTNF-α mutant, greater accumulation of regulatory T cells was found in the tumor site (15). TNFR deficiency in MDSCs results in a decrease in CXCR4 expression and the impaired recruitment of MDSCs to tumor tissue (27). mTNF-α/TNFR2 signaling also promotes MDSC survival *via* the upregulation of FLICE-inhibitory protein (c-FLIP) leading to the inhibition of caspase-8 activity (32). It has been identified that mTNF-α, rather than sTNF-α, can also enhance the immunosuppressive activity of MDSCs *via* TNFR2 (15). This action is related to the upregulation of arginase-1 and inducible NO synthase transcription, the promotion of NO, reactive oxygen species, IL-10, and TGF-β secretion, and the enhanced inhibition of lymphocyte proliferation. Upregulated expression of mTNF-α in 4T1 cells promotes tumor progression and angiogenesis in animal models and results in greater MDSC accumulation, increased NO, IL-10, and TGF-β levels, and poor lymphocyte infiltration. It has been demonstrated that p38 phosphorylation and NF-κB activation triggered by mTNF/TNFR2 are the most important mechanisms through which mTNF-α regulates MDSCs (15).

Although many studies have reported that mTNF/TNFR2 can enhance tumor progression by recruiting and activating MDSCs, Ardestani et al. (33) reported that mTNF-α-expressing tumor cells induced tumor-associated myeloid cell death. In a mouse model implanted with Lewis lung cancer cells or B16F10 melanoma cells expressing mTNF-α, tumor growth was decreased and related to significantly reduced tumor-associated myeloid cell infiltration. mTNF-α triggers ROS production in myeloid cells and induces necrotic cell death, but the mechanism by which mTNF-α induces ROS production needs to be further studied (34, 35). In another study, the mTNF-α-producing transformed tumor cell line HeLa was used as a “vaccine” to induce tumor rejection by stimulating macrophages to exert an anti-tumor effect; this strategy is believed to be a promising and safe cytokine gene therapy (36).

### mTNF-α/TNFR2 Regulates Survival, Apoptosis, and Metastasis of Tumor Cells through Forward and Reverse Signaling

In addition to regulating the accumulation and activation of immune cells in the tumor microenvironment, mTNF-α/TNFR2 also affects the survival, apoptosis, and metastasis of tumor cells directly. It has been reported that Raji cells, a human Burkitt lymphoma cell line, express both mTNF-α and TNFR2, which could mediate forward signaling or reverse signaling to induce cell death or survival *via* the NF-κB pathway (28). On the one hand, when mTNF-α acts as a ligand binding to TNFR2 on tumor cells, NF-κB activity is downregulated, which is followed by the subsequent inhibition of anti-apoptotic gene transcription, such as cIAP-1 and Fas-associated death domain-like IL-1β-converting enzyme-like inhibitory protein. On the other hand, when mTNF-α on tumor cells acts as a receptor to trigger reverse signaling, the activation of NF-κB is induced, and the production of anti-apoptotic proteins is further enhanced. Constitutive NF-κB activation causes Raji cells to be resistant to TNF-α-mediated cytotoxicity and sustains tumor cell survival (28, 37). These data indicate that there is a balance between forward and reverse signaling, but that reverse signaling is always dominant; consequently, this balance maintains constitutive NF-κB activation to sustain tumor cell viability (28). However, it was reported that the transfection of mTNF-α into the murine hepatic carcinoma cell line H22 upregulated Fas expression and induced tumor cell apoptosis *via* the Fas/FasL pathway. Moreover, mTNF-α inhibits CD44v3 expression to suppress tumor metastasis (38). We predict that in different types of tumor environment, the signaling pathways mediated by the mTNF-α/TNFR2 interaction are different, which may facilitate tumor survival or induce tumor cell apoptosis.

Since mTNF-α signaling influences survival and apoptosis of tumor cells directly, the expression of mTNF-α and its relationship with clinical characteristics was analyzed in cancer patients. It has been reported that mTNF-α expression is much higher in breast cancer compared with atypical dysplasia and hyperplasia, but mTNF-α is absent in normal breast tissue (37). In addition, the expression levels of mTNF-α are increased in acute leukemia (AL) and leukemia stem cells (LSCs). The high levels of mTNF-α expression in AL and LSCs correlate with poor risk stratification, extramedullary infiltration, and adverse clinical parameters (39). Targeting mTNF-α using a mAb inhibits leukemia cell growth and prevents the recurrence of leukemia in secondary serial transplantation into NOD-SCID mice (39). The *in vivo* and *in vitro* mAb experiments suggest that mTNF-α is a promising candidate for treating mTNF-α-positive tumors, especially in patients who are not sensitive to TNF antagonists (37, 39).

From the above findings, it can be concluded that different cell types are involved in the interaction between mTNF-α and TNFR2 in the tumor microenvironment and can influence tumor progression. On the one hand, the interaction facilitates tumor growth and progress. Tumor cells expressing mTNF-α recruit immune suppressive cells to the tumor microenvironment *via* TNFR2, which can suppress the anti-tumor immune response (15). Moreover, constitutive NF-κB activation triggered by reverse signaling protects Raji cells from sTNF-α-mediated cytotoxicity and sustains tumor cell survival (28). On the other hand, it has been reported that the mTNF-α expressed on Lewis lung cancer cells or B16F10 melanoma cells is related to reduced tumor-associated myeloid cell infiltration and decreased tumor growth (33). In addition, Raji cells can induce forward signaling in neighboring tumor cells through the interaction between mTNF-α and TNFR2, which leads to inhibition of NF-κB activation and mTNF-α-induced cell death. However, forward signaling is not dominant in Raji cells (28).

## Forward Signaling of mTNF-α/TNFR2

Unlike the pathways triggered by mTNF-α/TNFR1, the downstream signaling pathways triggered by TNFR2 have not been clearly clarified. TNFR2 has no enzymatic activity by itself, thus any signal transduction needs the recruitment of adaptor proteins (40, 41). Current studies suggest that the mTNF-α/TNFR2 interaction mediates most of the biological behaviors by recruiting TNFR2-associated factor (TRAF2) to bind to the cytoplasmic domain of TNFR2, which induces the activation of NF-κB, c-Jun N-terminal kinase (JNK), or AP-1 pathways (38, 42–44). TRAF proteins have seven members and they act as adaptor proteins between TNFR2 and the kinases involved in the activation of JNK and NF-κB (45, 46). Among the seven members, TRAF2 is the key mediator in the signaling pathways of TNFR2 (42). The intracellular region of TNFR2 contains several highly conserved sequences, including TRAF2-binding sites (comprising 402-SKEE-405 and amino acids 425–439) and module III (amino acids 338–379), which contains no TRAF2 binding sites but a region (amino acids 343–379) related to TRAF2 degradation (40, 43). Upon TNFR2 activation, TRAF2 translocates to a Triton X-100 insoluble compartment where TRAF2 is K48-linked ubiquitinated and finally degraded by the proteasome (42).

NF-κB is a transcriptional factor composed of homodimeric and heterodimeric complexes of related proteins from the Rel superfamily. The inhibitory subunit IκB-α stabilizes NF-κB (28, 47). IκB kinase (IKK) is activated upon the interaction between TNFR2 and TRAF2 (45, 46). Once phosphorylated by IKK, IκB-α is recognized for ubiquitination and is degraded by the proteosome, and the IκB-α/NF-κB complex can release NF-κB for translocation into the nucleus. In the nucleus, NF-κB binds to target gene promoters and induces the expression of these genes (47, 48). The activation of NF-κB can promote tumor cell survival and apoptosis resistance and MDSC activation (21, 27, 28). When TNFR1 and TNFR2 are co-expressed, TRAF2 degradation results in an enhanced TNFR1 cytotoxicity that is associated with the inhibition of NF-κB (43).

Jun N-terminal kinase is an important kinase that initiates a signaling pathway. JNK belongs to the mitogen-activated protein kinase family (49). In the context of TRAF2 interaction with TNFR2, JNK can be activated transiently by TRAF2 and prolonged activation can occur in a TRAF2-independent fashion. Module III (amino acids 338–379), which is a region on TNFR2 that contains no TRAF2 binding sites, is able to activate JNK in a TRAF2-independent manner (40, 43). Deletion of TRAF2-binding sites can eliminate TRAF2-induced NF-κB but not JNK activation. In the process of JNK activation, ASK1 interacting protein 1 (AIP1), which is an adaptor molecule, interacts with the amino acids 338–355 within module III to regulate the JNK pathway (50). The interaction between TRAF2 and TNFR2 induces both NF-κB and JNK activation to transmit proliferation signals. Then, TRAF2 degradation induced by TNFR2 contributes to inhibition of NF-κB and TRAF2-dependent JNK signaling, but TNFR2 is still able to activate a TRAF2-independent JNK pathway, which is responsible for TNFR2-dependent cell death in some cell types (51, 52). A recent study indicated that a new adaptor molecule known as aminopeptidase P3 (APP3, also known as XPNPEP3) was identified in the TNFR2 signaling complex. One of its two isoforms, mitochondrial APP3 (APP3m) is recruited to TNFR2 and induces TNF-TNFR2-dependent phosphorylation of JNK1 and JNK2, which exerts an anti-apoptotic function (53).

During the recruitment of MDSCs to tumor sites, mTNF-α/TNFR2 can activate both the NF-κB and p38 MAPK pathways (27). Research has demonstrated that both NF-κB and p38 MAPK mediate mTNF-induced MDSC activation. In this process, the level of p38 phosphorylation is significantly increased. Upon preincubation of MDSCs with a p38 MAPK inhibitor or NF-κB inhibitor, the immune suppressive function of MDSCs is abrogated. P38 MAPK activation is TRAF2-dependent as well. The interaction between mTNF-α and TNFR2 induces NF-κB and p38 MAPK activation and then CXCR4 expression increases, which contributes to the recruitment and activation of MDSCs (15, 27). Moreover, the p38 MAPK pathway regulates NF-κB transactivation *via* direct acetylation of p65 and is necessary for TNF-mediated NF-kB activation (54).

In addition to the signaling pathways mentioned above, the interaction between TRAF2 and TNFR2 induces the activation of the transcription factors AP-1 through MAPK3s (40). Moreover, tumor expressing mTNF-α can stimulate the Fas expression that mediates tumor cell apoptosis *via* the Fas/FasL pathway. However, the involved pathways need to be explored in greater detail (37).

## Reverse Signaling of mTNF-α/TNFR2

Increasing evidence suggests that mTNF-α can act as a receptor when interacting with sTNFR2, anti-TNF antibody, or TNFR2-expressing cells, thus activating intracellular signaling pathways. The outside-to-inside signaling mediated by mTNF-α is called reverse signaling. Reverse signaling is proven to be profoundly important in the activation of immune cells and apoptosis of macrophages (55, 56).

Take the interaction between monocytes and T cells in rheumatoid arthritis (RA) for example: TNFR2 on T cells behaves as a ligand and binds to mTNF-α on monocytes to trigger reverse signaling back into the monocytes, which contributes to the activation of monocytes. The reverse signaling mediated by mTNF-α/TNFR2 induces the activation of ERK1/2, which results in the dephosphorylation of the small cytoplasmic domain of mTNF-α and increases calcium concentrations. The reverse signaling transduced from mTNF-α to the nucleus activates monocytes to increase the production of TNF-α (57). It was reported that activated T cells in the synovial membrane of RA patients exhibiting a pathological phenotype that strongly induced the production of pro-inflammatory cytokines by monocytes through the interaction between mTNF-α and TNFR2 (57–59).

In addition, mTNF-α can mediate negative regulatory signaling that induces monocytes/macrophages to become resistant to inflammatory responses triggered by LPS (60). This negative regulatory response is mediated by the MAPK/ERK pathway (61). Pallai et al. (62) demonstrated that exposure of macrophages to LPS can induce the reverse signaling pathway *via* mTNF-α expressed on macrophages, after which, the reverse signaling activates the MAPK kinase (MKK) 4 pathway to induce TGF-β production. TGF-β then activates the ERK kinase pathway and mediates resistance to LPS-induced inflammation by inhibition of pro-inflammatory cytokines. In addition, the AKT pathways are also activated and are likely to act as a negative regulator of TGF-β production. However, the production of TGF-β mediated by mTNF-α reverse signaling is not a universal response. For example, when TNFR2-expressing T cells interact with mTNF-α-expressing monocytes/macrophages, the monocytes/macrophages are induced to produce TNF-α rather than TGF-β, as described above (57, 62). Because of the important role of TNF-α, anti-TNF agents have already been used in clinical treatment. TGF-β, which is induced by reverse signaling, plays an essential role in determining the therapeutic efficacy of TNF-α antagonists (63–69).

## Conclusion

Increasing evidence indicates that sTNF-α and mTNF-α play an essential role in the regulation of immune responses and tumor progression. In the tumor microenvironment, mTNF-α-expressing tumor cells contribute to the accumulation and activation of immunosuppressive cells to suppress the anti-tumor immune responses mediated by immune cells, which facilitates the growth and evasion of tumor cells. In addition, reverse signaling triggered by the interaction between mTNF-α and TNFR2 also plays a significant role in maintaining tumor cell survival and contributing to the metastasis of tumor cells. Targeting mTNF-α *via* mAbs is a promising strategy for treating mTNF-α-positive tumors. Notwithstanding its pro-tumor effect, the interaction between mTNF-α and TNFR2 can cause anti-tumor effects in indirect and direct ways. The mTNF-α/TNFR2 interaction suppresses tumor cells through the induction of tumor-associated myeloid cell death or the direct activation of the Fas/FasL pathway in tumor cells. The effects and mechanisms of mTNF-α/TNFR2 interaction in the tumor microenvironment, which include either regulating immunosuppressive cells or directly acting upon tumor cells, need to be further explored.

## Ethics Statement

This study was approved by Ethic committee of Tianjin Medical University.

## Author Contributions

YQ was responsible for the data collection and the draft of the manuscript. GZ gave assistance to finish the manuscript. HL came up to the idea of the research, conceived the manuscript, and modified it.

## Conflict of Interest Statement

The authors declare that the research was conducted in the absence of any commercial or financial relationships that could be construed as a potential conflict of interest.
